# Pro-inflammatory cytokine release from chicken peripheral blood mononuclear cells stimulated with lipopolysaccharide

**DOI:** 10.14202/vetworld.2022.885-889

**Published:** 2022-04-11

**Authors:** Jatuporn Rattanasrisomporn, Chananphat Tantikositruj, Anyarat Thiptara, Warangkana Kitpipit, Ittidet Wichianrat, Autchara Kayan, Chaiwat Boonkaewwan

**Affiliations:** 1 Department of Companion Animal Clinical Science, Faculty of Veterinary Medicine, Kasetsart University, Bangkok 10900, Thailand; 2 Department of Animal Science, Kasetsart University, Bangkok 10900, Thailand; 3 Department of Livestock Development, Veterinary Research and Development Center (Upper Southern Region), Nakhon Si Thammarat 80110, Thailand; 4 Akkhraratchakumari Veterinary College, Walailak University, Nakhon Si Thammarat 80160, Thailand; 5 One Health Research Center, Walailak University, Nakhon Si Thammarat 80160, Thailand; 6 Food Technology and Innovation Center of Excellence, Walailak University, Nakhon Si Thammarat, 80160, Thailand

**Keywords:** chicken, interleukin-1β, interleukin-6, peripheral blood mononuclear cell, tumor necrosis factor-α

## Abstract

**Background and Aim:**

The principal cytokines released by the host on infection include pro-inflammatory cytokines such as interleukin (IL)-1, IL-6, and tumor necrosis factor (TNF). These cytokines were regarded as regulators of the host’s response to infection. This study aimed to determine the release of pro-inflammatory cytokines from chicken peripheral blood mononuclear cells (PBMCs) following lipopolysaccharide (LPS) stimulation.

**Materials and Methods:**

Blood samples were collected from six Betong chickens. To isolate PBMCs, density gradient centrifugation was utilized. PBMC culture in RPMI1640 with 10% fetal bovine serum was stimulated with various concentrations of LPS (0, 0.01, 0.1, and 1.0 μg/mL). The production of TNF-α, IL-1β, and IL-6 was determined using an enzyme-linked immunosorbent assay.

**Results:**

When the PBMCs were cultured for 24 h with varying doses of LPS, there was no significant variation in cell viability. TNF-α, IL-1β, and IL-6 levels were measured in Betong chicken PBMC. The release of these cytokines increased considerably as LPS concentration (0.01-1 μg/mL) increased (p<0.05).

**Conclusion:**

*In vitro* studies of the chicken immune response, notably the release of pro-inflammatory cytokines, can be conducted using PBMCs obtained from chicken blood.

## Introduction

Inflammation is a host’s initial immune response to pathogens, mediated by immune cells and associated cytokines. Pathogenic bacteria and other infectious agents can directly activate monocytes or macrophages, initiating a cytokine cascade in the inflammatory process and the immunological response. As with mammals, many pathogen- or damage-associated molecular patterns stimulate the inflammatory response in birds through their interaction with cellular and soluble pattern recognition receptors [1,2]. The Gram-negative bacterial cell wall, in particular lipopolysaccharide (LPS), can induce monocytes and macrophages to release pro-inflammatory cytokines. Tumor necrosis factor (TNF)-α, interleukin (IL)-1β, and IL-6 have all been identified as significant inflammatory mediators and have been implicated in the development of a variety of inflammatory diseases [3-5].

It has been demonstrated that toll-like receptor (TLR)4, which is expressed in chicken peripheral blood mononuclear cells (PBMCs) [6], is required for the immune cell to respond to LPS stimulation by releasing pro-inflammatory cytokines. This ligand-receptor interaction induces macrophages to synthesize and release vasoactive mediators, cytokines (IL-1β, IL-6, and TNF-α), and chemokines [7-9]. In addition, the interference in the production of TNF-α, IL-1β, and IL-6 can be employed as criteria to evaluate the anti-inflammatory effects of natural products [10].

The PBMC population is mainly composed of lymphocytes (T cells, B cells, and natural killer cells), monocytes, and a trace of other immune cells such as dendritic cells. PBMCs are frequently used in immunological or molecular investigations [11]. A previous study found that LPS-induced maturation increased the level of pro-inflammatory cytokine and chemokine expression in chicken bone marrow and monocyte-derived dendritic cells [12].

However, there are no data on pro-inflammatory cytokine release from chicken PBMC. Thus, this study aimed to examine the release of pro-inflammatory cytokines and monitor their production using an *in vitro* model of LPS-stimulated chicken PBMC.

## Materials and Methods

### Ethical approval

The Animal Ethics Committee at Kasetsart University authorized all techniques utilized in this investigation (ACKU61-AGR-009).

### Study period and location

The study was conducted from November 2018 to March 2020. The Vajokkasikij Chicken Farm at Kasetsart University contributed chickens for this study. The Department of Animal Science at Kasetsart University’s Faculty of Agriculture and the Department of Companion Animal Clinical Science at the Faculty of Veterinary Medicine processed the samples.

### Animals and blood collection

Whole blood was collected from six Betong chicks aged 14-18 weeks. Three milliliters of blood were drawn from the wing vein into an anticoagulant tube containing ethylenediaminetetraacetic acid. Each blood sample was partitioned into two sections. To ensure that the blood was obtained from healthy chicks, the initial fraction (0.5 mL) was employed to ascertain the blood’s hematological (complete blood count) level. The remaining volume (2.5 mL) was used to isolate PBMCs.

### PBMC isolation and culture

The PBMCs were isolated from blood samples by gently layering 2.5 mL of blood over 3 mL of Histopaque solution (Sigma-Aldrich, St. Louis, MO) and centrifuging at 400× *g* for 30 min. By centrifugation at 400 × g for 5 min, the white band of mononuclear cells was collected and washed 3 times with RPMI 1640 culture media. The PBMCs were suspended in RPMI 1640 medium (25 mM HEPES, 2 mM L-glutamine, 10% heat-inactivated fetal calf serum, penicillin [100 U/mL], and streptomycin [100 g/mL]) before being adjusted to a concentration of 2×10^6^ cells/mL. The trypan blue dye exclusion technique was used to measure the viability of cells. Viability >90% was accepted for the LPS activation experiment.

In a 96-well plate, PBMCs were divided into four groups: (1) PBMC alone (control), (2) PBMC with 0.01 mg/mL LPS, (3) PBMC with 0.01 mg/mL LPS, and (4) PBMC with 1 mg/mL LPS. For 24 h, all four sets of PBMCs were cultured in a humidified environment containing 5% CO_2_ at 41°C. Each well’s culture medium was collected and stored at −20°C for TNF-α, IL-1β, and IL-6 measurement.

### Cell viability assay

The trypan blue dye exclusion technique was performed to determine the viability of PBMCs after 24 h of culture with different doses of LPS (0.01-1 μg/mL) in a humidified atmosphere containing 5% CO_2_ at 41°C. Cells were collected using a hemocytometer, and viable cells were counted.

### Pro-inflammatory cytokines measurement

On the day of the experiment, the culture medium was thawed from the freezer (−20°C) and prepared for TNF-α, IL-1β, and IL-6 quantification using an enzyme immunosorbent assay kit (Cloud-Clone Corp., USA), according to the manufacturer’s instructions.

### Statistical analysis

Prism 5 software (GraphPad Software, USA) was used to analyze the data, presented as mean±standard deviation. To determine the statistical significance of the effect of different LPS concentrations on TNF-α, IL-1β, and IL-6 levels in chicken PBMC, one-way analysis of variance and Student–Newman–Keuls method were used. Statistical significance was defined as p<0.05.

## Results

### Cell viability

**Figure-1:**
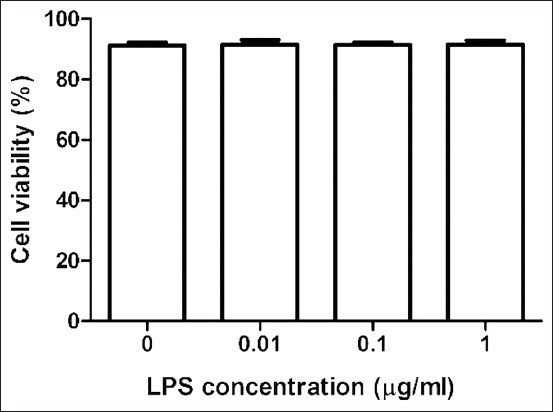
The viability of peripheral blood mononuclear cells after 24 h culture with different doses of lipopolysaccharide (0.01-1 μg/mL) in a humidified atmosphere containing 5% CO_2_ at 41°C. Data are expressed as the mean±standard deviation of six independent experiments.

PBMC isolated from Betong chicken blood samples demonstrated high cell viability (94%), and all hematological parameters were within the normal range (data not shown). The cytotoxic effects of LPS on PBMC were determined to corroborate the non-toxic concentration of LPS used to measure pro-inflammatory cytokines release. As illustrated in Figure-1, when PBMCs were grown with varying concentrations of LPS (0, 0.01, 0.1, and 1 μg/mL) for 24 h, there was no significant difference in PBMC viability (91.30.9, 91.5*±*1.6, 91.5*±*0.8, and 91.7*±*1.3).

### Effect of LPS on TNF-α release

**Figure-2:**
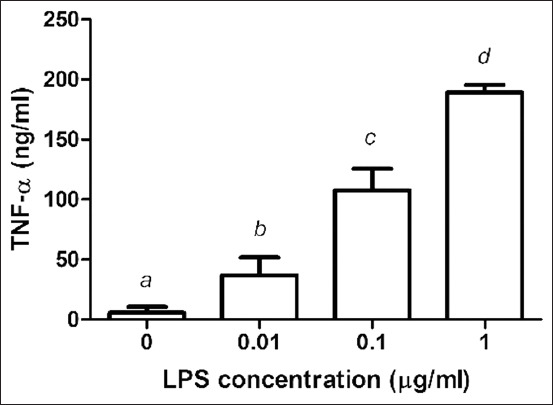
Effects of lipopolysaccharide (LPS) on the production of tumor necrosis factor-α (TNF-α) in peripheral blood mononuclear cells (PBMCs). PBMCs were cultured with LPS (0.01-1 μg/mL). Data are expressed as the mean±standard deviation of six independent experiments. ^a,b,c,d,e,f^Statistically significant difference in TNF-α release in each column (p<0.05).

As shown in Figure-2, the stimulation of PBMC with LPS at concentrations of 0, 0.01, 0.1, and 1 μg/mL resulted in a dose-dependent increase in the TNF-α levels to 5.74*±*5.03, 37.05*±*15.06, 108.93*±*15.36, and 189.85*±*4.96 ng/mL, respectively.

### Effect of LPS on IL-1β release

**Figure-3:**
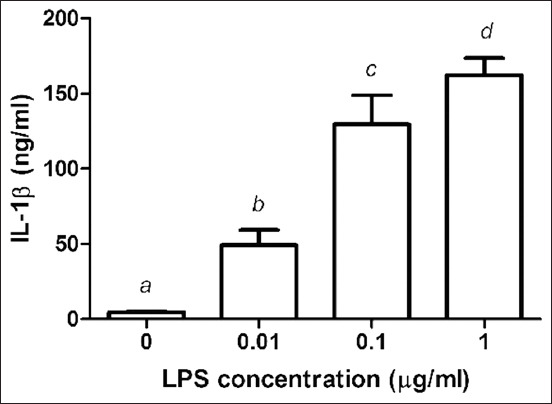
Effects of lipopolysaccharide (LPS) on the production of interleukin (IL)-1β from chicken peripheral blood mononuclear cells (PBMCs). PBMCs were cultured with LPS (0.01-1 μg/mL). Data are expressed as the mean±standard deviation of six independent experiments. ^a,b,c,d,e,f^Statistically significant difference in IL-1β release in each column (p<0.05).

The release of IL-1β is indicated in Figure-3. The stimulation of PBMC with LPS at doses of 0.01, 0.1, and 1 μg/mL resulted in a dose-dependent increase in the IL-1β levels to 5.74*±*5.03, 37.05*±*15.06, 108.93*±*15.36, and 189.85*±*4.96ng/mL, respectively.

### Effect of LPS on IL-6 release

**Figure-4:**
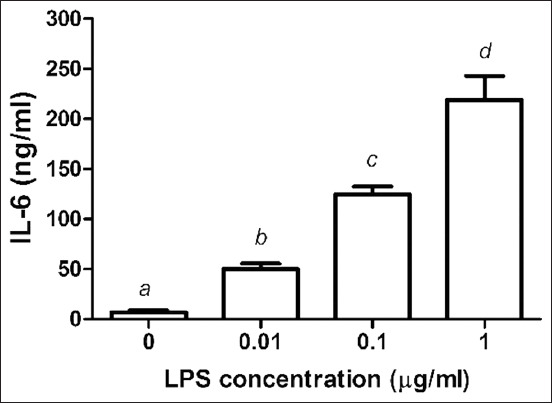
Effects of lipopolysaccharide (LPS) on the production of interleukin (IL)-6 from chicken peripheral blood mononuclear cells (PBMCs). PBMCs were cultured with LPS (0.01-1 μg/mL). Data are expressed as the mean±standard deviation of six independent experiments. ^a,b,c,d,e,f^Statistically significant difference in IL-6 release in each column (p<0.05).

Figure-4 depicts the release of IL-6. When LPS was added to PBMC at concentrations of 0.01, 0.1, and 1 μg/mL, the level of IL-6 increased dose-dependently to 6.57*±*2.41, 50.23*±*5.29, 124.41*±*8.20, and 219.18*±*23.64 ng/mL, respectively.

## Discussion

Betong is a breed of chicken that originated in Thailand. While Betong production has many advantages, including rapid development, large revenue, and easy market access, it also has several negatives, including difficulty raising due to a low tolerance for the rainy season and a high cost of breeders. The primary causes of death in the Betong chicken are disease and accidents [13]. Chicken feces, feathers, germs, and fungi, as well as gaseous pollutants such as ammonia, hydrogen sulfide, and carbon dioxide, cause high contamination in chicken farm environments. Poultry farm dust contains high levels of endotoxin, also known as LPS [14,15]. As a result, better understanding their immunological response should be one of the answers to advancing Betong manufacturing.

Before initiating an *in vitro* inquiry, it is critical to determine cell viability because this serves as an early indicator of cell quality; viability values more than or equal to 95% are considered excellent [16]. The viability of the PBMCs employed in this investigation was determined to be very high (>94%). In addition, 24 h of culturing PBMC with various doses of LPS does not influence their vitality. As a result, all cytokines released in this study are a result of LPS stimulation.

When chickens are exposed to various stimuli, including pathogens or allergens, their body responds by secreting cytokines, which stimulate cells to take protective measures against other cells and tissues. It frequently results in inflammation, such as sepsis, with the primary cytokines secreted being IL-1β, IL-6, and TNF-α [17-19]. On the other hand, pro-inflammatory cytokines are not secreted under normal settings but are released in response to immune system stimulation [20].

Bacterial LPS is frequently used in research to induce inflammation and release pro-inflammatory cytokines and inflammatory mediators. LPS covalently links to TLR4 to form the TLR4/myeloid differentiation protein-2 complex. TLR4 signaling and its downstream molecules, along with certain cytokines, play a key role in chickens [21-23]. According to cytokine expression studies, peripheral blood monocyte-derived dendritic cells treated for 24 h with LPS (1 μg/mL) expressed the highest quantities of IL-1 and IL-6 [12]. After 24 h of LPS stimulation of PBMCs demonstrated that TNF-α, IL-1β, and IL-6 secretions increased with increasing LPS concentrations, with LPS at 1 mg/mL concentration eliciting the greatest release of pro-inflammatory cytokines from PBMCs.

## Conclusion

Improved understanding of the chicken’s immunological response should be one of the solutions to enhancing chicken production. Chicken blood PBMCs can be used to investigate *in vitro* chicken immune response, specifically the release of pro-inflammatory cytokines in response to LPS.

## Authors’ Contributions

JR and CT: Collected the samples and conducted the study. CT: Drafted the manuscript. AK and IW: Technical guidance and supervision. AT and WK: Statistical analysis. CB: Designed the study, collected the data, and reviewed the manuscript. All authors read and approved the final manuscript.
